# Identifying hidden coalitions in the US House of Representatives by optimally partitioning signed networks based on generalized balance

**DOI:** 10.1038/s41598-021-98139-w

**Published:** 2021-10-07

**Authors:** Samin Aref, Zachary P. Neal

**Affiliations:** 1 Max Planck Institute for Demographic Research, 18057 Rostock, Germany; 2 Department of Mechanical and Industrial Engineering, University of Toronto, Toronto, ON M5S3G8 Canada; 3 Department of Psychology, Michigan State University, East Lansing, MI 48824 USA

**Keywords:** Applied mathematics, Computational science, Computer science

## Abstract

In network science, identifying optimal partitions of a signed network into internally cohesive and mutually divisive clusters based on generalized balance theory is computationally challenging. We reformulate and generalize two binary linear programming models that tackle this challenge, demonstrating their practicality by applying them to partition signed networks of collaboration and opposition in the US House of Representatives. These models guarantee a globally optimal network partition and can be practically applied to signed networks containing up to 30,000 edges. In the US House context, we find that a three-cluster partition is better than a conventional two-cluster partition, where the otherwise hidden third coalition is composed of highly effective legislators who are ideologically aligned with the majority party.

## Introduction

Signed networks, in which nodes can be connected by positive or negative ties, occur in many contexts. To identify communities in signed networks, it is often useful to put the nodes into clusters so that most positive ties are within clusters, while most negative ties are between clusters. Identifying clusters of nodes that optimally meet these criteria is computationally challenging, but we present practical methods for doing so. Applying these new global optimization methods to signed networks of the US House of Representatives shows that legislators are actually organized into three coalitions whose ideological composition offers new insights on the otherwise obscured interplay between partisanship and legislative effectiveness.

Signed networks are studied in a diverse range of contexts in both the natural^1–3^ and social^4–7^ sciences. Across these contexts, it is often of interest to identify clusters of nodes that are internally cohesive and mutually divisive, and thus partially satisfy the conditions of *generalized balance*^8–11^. Recent computational work on signed network analysis has focused on determining the network’s level of balance in general^12–15^, and in the context of signed graphs with node attributes^16,17^. However, although optimization-based methods exist for estimating a network’s level of balance^18^ by heuristically partitioning it into $k=2$ clusters^13^ or computing its exact level of balance by optimally partitioning it into $k=2$ clusters^2,19,20^, identifying an optimal partition of nodes into $k\ge 2$ clusters that corresponds to the network’s level of *k*-*balance* (a.k.a. *weak balance*, generalized balance, and *clusterability*^8^) has remained a challenge. This computational challenge involves solving fundamental non-deterministic polynomially acceptable hard (NP-hard) graph optimization problems to global optimality^19,21–23^.

A common misconception about solving NP-hard optimization problems is that they can be addressed using “only heuristic methods”^24^. Previous work in this area has used a modified concept of network *modularity* to incorporate signed edges into a modularity maximization procedure^24,25^. They used a *tabu search* heuristic algorithm on a signed network with 1131 edges^25^ and used a *simulated annealing* heuristic algorithm on a signed network with 2517 edges^24^, in each case settling for sub-optimal partitions whose distance from optimality remains unknown. Unlike modularity, the concept of *frustration*^15,26^ requires no modification for application in signed networks because it originates from Ising models of atomic magnets in which couplings of opposite nature exist^27^ which are analogous to signed ties. Using frustration and two mathematical optimization models, we propose and demonstrate a general method for finding a *globally optimal* partition of signed networks into $k\ge 2$ clusters.

Identifying an optimal partition of nodes into internally cohesive and mutually divisive clusters involves two computational challenges. The first challenge is finding a *k*-partition of a signed network, placing nodes into *k* clusters that minimize intra-cluster negative and inter-cluster positive edges (*frustrated edges*), where *k* is selected in advance^19^. A second challenge is finding the smallest number of clusters $k^*_\text {min}$ that minimizes frustrated edges among all partitions across all values of *k*. These challenges are unique from, but conceptually analogous to related challenges in community detection in unsigned networks: It is difficult to find a modularity maximizing partition into a specific number of clusters, but even harder to find the modularity maximizing partition into any number of clusters^28^. We solve the first challenge by generalizing a mathematical programming model for finding an optimal 2-partition^15,19^ and introducing a generalized model to find optimal *k*-partitions. Then, we tackle the second challenge by reformulating another mathematical model^22^ for non-complete graphs and solving it without providing the number of clusters.

We demonstrate the practicality of these methods, and illustrate how they can generate novel insights, by applying them to signed networks of political collaboration and opposition in the US House of Representatives from 1981 to 2018. Research on and descriptions of the US House usually place legislators into clusters defined by legislators’ political party affiliations. However, reliance on a simple binary attribute risks oversimplifying this complex system because it ignores information about the positive and negative interactions between individual legislators. We explore whether placing legislators into optimal clusters defined by their interactions, rather than simply by their parties, better captures the coalitional structure of the chamber. We find that the best fitting parsimonious solution places legislators into three clusters characterized by a large liberal coalition, a large conservative coalition, and a smaller ideologically fluid coalition. Interestingly, we find that members of this ideologically fluid third coalition are substantially more effective at passing legislation than members of either dominant coalition. These findings suggest that, although political parties are clearly influential in US politics, some of the heavy lifting in the US House is done by a small splinter coalition of highly effective legislators who are ideologically aligned, but not necessarily collaborating, with members of the majority party’s core.

## Partitioning signed networks

In this section, after introducing notions of *k*-balance and signed networks, we propose two related mathematical models. The first model finds a globally optimal partition of nodes into exactly *k* clusters. The second model finds a globally optimal partition across all possible partitions. When used together, these models also provide the smallest number of clusters, $k^*_\text {min}$according to generalized balance.

### Preliminaries

A *signed network* is an undirected simple graph with positive and negative signs on the edges usually denoted as $G = (V,E,\sigma )$ where *V* and *E* are the sets of nodes and edges respectively, and $\sigma$ is the sign function $\sigma : E\rightarrow \{-1,+1\}$. Signed graph *G* contains $|V|=n$ nodes and its symmetric signed adjacency matrix is denoted by $\mathbf{A}$. The set *E* of edges contains $m^-$ negative edges and $m^+$ positive edges adding up to a total of $|E|=m=m^+ + m^-$ undirected signed edges. An edge with endpoints *i* and *j* is represented by (*i*, *j*) such that $i<j$. Given a signed graph $G=(V,E,\sigma )$, a *k-partition* is a division of the set of nodes *V* into *k* non-empty subsets $V_1,V_2,\dots ,V_k$ such that $V_i \cap V_j=\varnothing \forall i \ne j$ and $\cup _{i=1}^k V_i=V$ (i.e. every node belongs to exactly one subset).

Balance theory was conceptualized in the 1940s in the context of social psychology^29^, recast in graph theoretic terms in the 1950s^18^, and generalized in the 1960s^8^. Whereas classic balance holds that a signed network can be partitioned into up to two clusters^18^, generalized balance holds that it can be partitioned into any number of clusters. Generalized balance theory allows a more flexible structural decomposition of networked systems, which in turn offers a more nuanced view of polarization in social and political systems^30–32^. According to generalized balance theory, a signed network is *k*-*balanced* (i.e. *clusterable*) if its set of nodes can be partitioned into *k* clusters (or “coalitions”^33^) such that each positive edge joins nodes belonging to the same cluster, and each negative edge joins nodes belonging to different clusters^8^. Edges that fail to meet these criteria (i.e. a negative edge within a cluster, or positive edge between clusters) are called *frustrated* edges under that partition.

Generalized balance in empirical signed networks can be analyzed by measuring their distance to clusterability^9,11,19^. The distance of a given network *G* to clusterability can be quantified as the minimum number of frustrated edges among all possible partitions into *k* clusters [^11^, $k\text{-} \mathrm{clusterability\,index,} \,C_k(G)$], or the minimum number of frustrated edges among all possible partitions with any number of clusters $1\le k \le n$ [^9^, *clusterability index, *$C(G)$]. Obtaining these measures require intensive computation and are NP-hard^21^.

**Figure 1:**
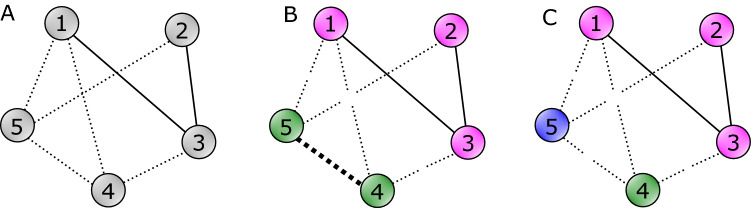
(**A**) An example signed network. (**B**) Evaluating classic balance via bi-partitioning involves finding the optimal 2-partition $\{\{1,2,3\},\{4,5\}\}$ and the edge (4, 5) which is frustrated under it. (**C**) Evaluating generalized balance and clusterability via *k*-partitioning leads to the optimal 3-partition $\{\{1,2,3\},\{4\},\{5\}\}$ which reduces the frustrated edges to 0.

Figure 1A shows an example signed network with five negative edges (dotted lines) and two positive edges (solid lines). The signed network can be optimally partitioned into two clusters based on classic balance (B), or three clusters based on generalized balance (C). The classic approach leads to the 2-partition $\{\{1,2,3\},\{4,5\}\}$ (shown by green and purple colors in Fig. 1B) which minimizes the total number of intra-cluster negative and inter-cluster positive edges to $C_2(G)=1$. The generalized approach (Fig. 1C), leads to the 3-partition $\{\{1,2,3\},\{4\},\{5\}\}$ which satisfies the conditions of generalized balance ($C(G)=C_3(G)=0$).

### Finding an optimal *k*-partition and the *k*-clusterability index

We formulate an optimization model that computes the *k*-clusterability index of an input signed network in its optimal objective function. In a given feasible solution of the optimization problem, each node belongs to one of a set of *k* clusters $C=\{1, 2, \dots , k\}$. The binary decision variable $x_{ic}$ takes the value 1 if node $i \in V$ belongs to cluster $c\in C$ (and $x_{ic}=0$ otherwise).

We consider that a positive edge $(i,j) \in E^+$ is frustrated (indicated by $f_{ij}=1$) if its endpoints *i* and *j* are in different clusters; otherwise it is not frustrated (indicated by $f_{ij}=0$). A negative edge $(i,j) \in E^-$ is frustrated (indicated by $f_{ij}=1$) if its endpoints *i* and *j* are in the same cluster; otherwise it is not frustrated (indicated by $f_{ij}=0$).

Using the binary decision variable $x_{ic}$, we formulate the process of finding an optimal *k*-partition and computing the *k*-clusterability index as the binary linear programming model in Eq. (1). The model in Eq. (1) is an extension of a model based on classic balance which provides an optimal 2-partition and computes the 2-clusterability index (a.k.a. the frustration index) of a signed network^15,19^.

1 $$\begin{aligned} \begin{aligned} \min \sum _{(i,j) \in E}&f_{ij} \\ \text {s.t.} \quad \sum _{c \in C} x_{ic}&= 1 \quad \forall i \in V \\ f_{ij}&\ge x_{ic} - x_{jc} \quad \forall (i,j) \in E^+, ~\forall c \in C \\ f_{ij}&\ge x_{ic} + x_{jc} -1 \quad \forall (i,j) \in E^-, ~\forall c \in C \\ x_{ic}&\in \{0,1\} \quad \forall i \in V, ~\forall c \in C \\ f_{ij}&\in \{0,1\} \quad \forall (i,j) \in E \end{aligned} \end{aligned}$$

The objective function in Eq. (1) computes the minimum number of frustrated edges among all *k*-partitions. The first set of constraints in Eq. (1) ensures that each node belongs precisely to one cluster. The second and third sets of constraints formulate the relationship between frustration of an edge (left-hand side) and the cluster membership of the endpoints of that edge (right-hand side) respectively for positive edges and negative edges. Refer to the Supplementary Information for more details and an illustrative numerical example on how the *k*-partitioning model in Eq. (1) works.

### Finding an optimal partition and the clusterability index

The more general problem of finding an optimal partition without specifying *k* and computing the clusterability index of a signed network *G* is known as the Correlation Clustering problem^21^ (and the Clique Partitioning problem if the graph is complete^34^). We reformulate the mathematical model initially proposed in^22^ which is defined in the context of complete graphs and widely used in the literature^35–38^ as follows. For every pair of nodes $i,j,i<j$, we define the binary decision variable $y_{ij}$ which takes the value 1 if *i* and *j* belong to the same cluster and takes the value 0 otherwise.

2 $$\begin{aligned} \begin{aligned} \min \sum _{(i,j) \in E}&a_{ij}((a_{ij}+1)/2) - a_{ij}y_{ij} \\ \text {s.t.} \quad y_{ij} + y_{ik}&\ge 1 + y_{jk} \quad \forall (i,j,k) \in T \\ y_{ij} + y_{jk}&\ge 1 + y_{ik} \quad \forall (i,j,k) \in T \\ y_{ik} + y_{jk}&\ge 1 + y_{ij} \quad \forall (i,j,k) \in T \\ y_{ij}&\in \{0,1\} \quad \forall i \in V, ~j \in V ,~i<j \\ \end{aligned} \end{aligned}$$

The model in Eq. (2) uses these binary variables to count the frustrated edges in the objective function. In Eq. (2), the term $a_{ij}$ represents the entry of the input graph’s adjacency matrix $\mathbf{A}$ associated with the pair of nodes $i,j \in V$. To efficiently handle possibly non-complete graphs, we use the set *T* for the constraints of the model in Eq. (2). $T=\{(i,j,k)\in V^3 \mid |a{_i}{_j}|+|a{_i}{_k}|+|a{_j}{_k}| \ge 2, i<j<k \}$ denotes the set of all connected triads (node triples connected by at least two edges) in *G*. Refer to the Supplementary Information for more details and an illustrative numerical example on how the partitioning model in Eq. (2) works.

Although we use both models in Eqs. (1)–(2), they are not necessarily dependent. Under the assumption that $k<<<n$, our proposed model in Eq. (1) is less computationally intensive than the model proposed by^22^, which we have reformulated in Eq. (2). Despite similar scaling of the number of variables with ${\mathcal {O}}(n^2)$, constraints of (1) have a quadratic growth, ${\mathcal {O}}(n^2)$, while constraints of (2) have a cubic growth, ${\mathcal {O}}(n^3)$.

These models can be used for optimally partitioning any signed network into internally cohesive and mutually divisive clusters based on generalized balance. However, it is important to note that they can yield a multiplicity of optimal solutions, that is, they do not necessarily yield a single unique partition because multiple optimal solutions may exist (see the Supplementary Information for more details). Despite this potential multiplicity, these models offer two kinds of advantages over existing methods with similar goals. First, unlike heuristic partitioning methods that can provide locally optimal partitions^24^, the partitions identified by these models come with a *guarantee* of *global optimality* that means no better partition exists. Second, unlike other optimal partitioning methods that have been applied to small^23,35^ or complete^21^ signed networks, these models can be practically solved even for networks of considerable size and order, and for networks that are not complete, which are typical in social contexts. In the next section, we demonstrate their practicality using networks with up to 30,000 edges. We solve the optimization models in Eqs. (1) and (2) to global optimality using *Gurobi* solver (version 9.1)^39^ on a virtual machine with 32 Intel Xeon CPU E7-8890 v3 @ 2.50 GHz processors running 64-bit Microsoft Windows Server 2019 R2 Standard.

## Partitioning the US House networks

In the previous section, we generalized one model and reformulated another model that provide globally optimal partitions of a signed network according to generalized balance. In this section, we show that they are computationally feasible and can be solved in a practical amount of time. To illustrate their practicality, we apply them to 19 networks varying in size, density, and structure that represent political collaborations and oppositions in the US House of Representatives in different eras. Although these networks are not ‘large’ compared to some networks ($n \sim 445$, $4954 \le m \le 31936$), they are large by comparison to the size of signed networks for which globally optimal partitions have been obtained before^23,35,40^.

### Optimal coalitions

We compare several ways to partition US House legislators into clusters or “coalitions”^33^, with the goal of determining the optimal number and the composition of these coalitions. The fitness of a given partition is indicated by its associated number of frustrated edges. The conventional method is to partition legislators into coalitions based on their party affiliations, while here we also explore partitioning legislators into coalitions by applying the optimization models in Eqs. (1)–(2) to signed networks of their collaborations and oppositions. Throughout our application of these models in the US House context, we use the term “coalition” to refer to the clusters of legislators within a partition, however the partition is obtained, not only because it is commonly used in political contexts, but also because it was the term suggested for signed network partitions by Harary and Kabell^33^. Legislators’ memberships in these coalitions depend on either an attribute (e.g. their political party affiliation) or the solution to the models in Eqs. (1)–(2), but does not necessarily imply their cohesion with other members of the same coalition.

**Figure 2:**
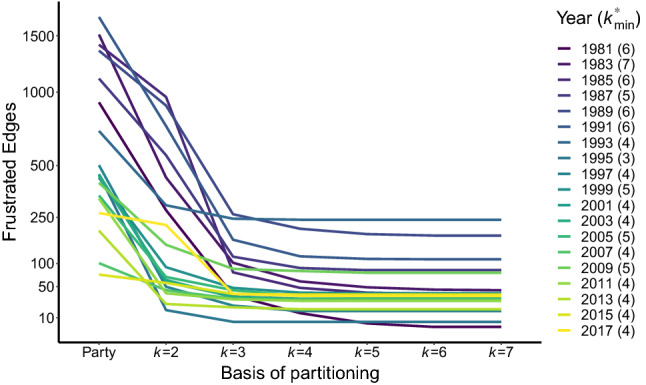
Number of frustrated edges (y-axis) of signed US House networks partitioned using different criteria (x-axis). Each line represents a single network, corresponding to a session of the US House starting in the given year. Fewer frustrated edges indicate that the partition is more consistent with the ties of collaboration and opposition between legislators.

Figure 2 illustrates the number of frustrated edges (y-axis) for partitions based on party affiliations and optimal *k*-partitions for $k \in \{2,3,\dots ,7\}$ (x-axis) in signed US House networks (see *SI* Table S1). The number of frustrated edges for a party-based partition (denoted by $C_{\text {party}}(G)$) is considerably larger than that of an optimal 2-partition. This implies that defining coalitions simply in terms of legislators’ party affiliations leads to many frustrated edges, and therefore to a poor description of the coalition structure of the chamber. The number of frustrated edges decreases further from $k=2$ to $k=3$, which implies that defining coalitions in terms of classic balance still leads to many frustrated edges and thus a poorer fit than defining coalitions in terms of generalized balance. For $k>3$ there is only marginal decline, and then stagnation, in the number of frustrated edges. Substantively, these results suggest that the signed US House networks are better described by a partition into $k>2$ coalitions than by a more conventional partition into only two coalitions^20^.

Figure 2 also reveals the changes over different eras of the House (e.g. sessions with start years 1981−1993 in darker blue-purple shades and 2003−2017 sessions in lighter green-yellow shades). Party-based partitions offer a better fit (i.e. fewer frustrated edges) in recent sessions than in earlier sessions due to increases in partisanship^5,20,41^. However, despite changes in the level of partisanship over time, for every session $C_{\text {party}}(G)> C_2(G) > C_3(G)$.

Because the results from Fig. 2 only cover a small range of *k*, a natural question is whether the fit could be improved further by using larger values of *k*. Finding the answer is not practically feasible using only the model in Eq. (1). Therefore, we solve the model in Eq. (2) to find the minimum number of frustrated edges, *C*(*G*), across all possible partitions for all possible values of $1\le k \le n$. By juxtaposing $C_k(G)$ from the model in Eq. (1) and the values of *C*(*G*) from the model in Eq. (2), we determine whether the low-points observed in Fig. 2 represent the clusterability indices *C*(*G*) of the networks or the number of frustrated edges could decline further for $k>7$.

Through this comparison, we verify that further decline in the frustrated edges is not possible because among all 19 networks, $C(G) = C_k(G)$ at $k\le 7$. The legend of Fig. 2 shows for each network the exact *point of stagnation* $k^*_{\text {min}}$, which is the smallest number of clusters that minimizes the *k*-clusterability index across all values of *k*: $k^*_{\text {min}}=\text {arg} \min _{1 \le i \le n} C_i(G)$.

### Coalition ideology

**Figure 3:**
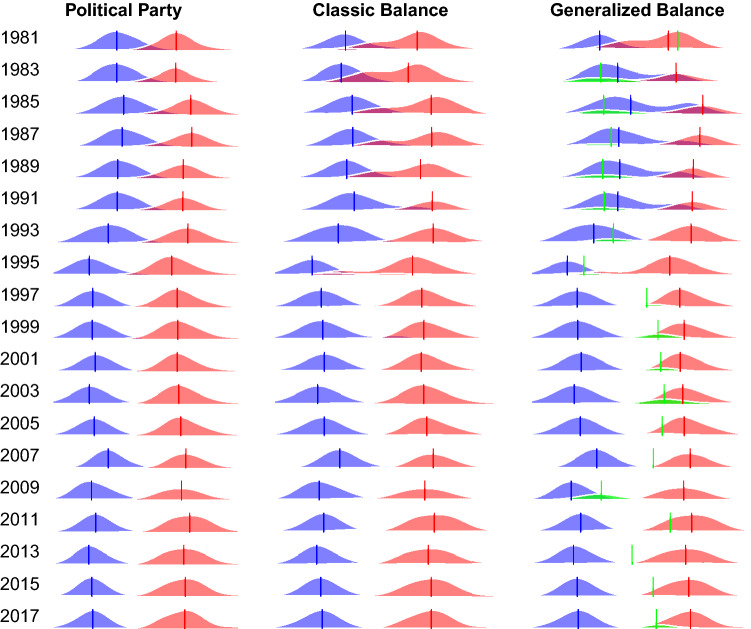
Distribution of coalition members’ ideology in the US House of Representatives. Blue (red) curves indicate the ideologies of Democrats (Republicans) in the left column and that of the dominant liberal (conservative) coalitions in the center and right columns. In the right column, green curves indicate the ideologies of members of the smallest coalition.

Having identified several ways to assign legislators to coalitions in the US House, including optimal *k*-partitions and optimal partitions, we now examine the ideological compositions of coalitions defined from three perspectives: party, classic balance ($k=2$), and generalized balance ($k=3$). Although we found that $3 \le k^*_{\text {min}} \le 7$, in the remaining substantive analyses we focus on the 3-partitions in the generalized balance case because $k>3$ offers only small improvements in fit and therefore $k=3$ offers a reasonable trade-off between fit and parsimony (see *SI* Figures S1 and S2). Figure 3 displays the distribution of coalition members’ ideology, for each method of defining coalitions (see *SI* Table S2). Coalitions with left-leaning liberal ideologies are shaded blue, while coalitions with right-leaning conservative ideologies are shaded in red; the solid vertical lines indicate a coalition’s median ideology.

Partitioning legislators into coalitions based on their political party affiliations (Fig. 3, left column) is the conventional approach in political science, and here displays the familiar pattern of increasing ideological polarization. Partitioning legislators based on classic balance (Fig. 3, center column) offers a more data-driven classification because legislators’ coalition memberships are based on their collaborative and oppositional interactions, but is still restrictive because it allows a maximum of two coalitions. The classic balance coalitions display similar ideological distributions to those based on political party: Increasing liberal-conservative ideological polarization.

Partitioning legislators into 3 coalitions based on generalized balance (Fig. 3, right column) also offers a data-driven classification, but allows more nuance. Like the other partitions, the generalized balance partition is characterized by a large liberal coalition and a large conservative coalition that diverge over time. However, it also includes a smaller and ideologically fluid coalition shaded in green. In the 435-member chamber, this ‘third coalition’ ranges in size from only 4 members in the 113th session (2013) to 69 members in the 111th session (2009). It also ranges in ideology from very liberal in the 98th–102nd sessions (1983–1991), to center-left in the 103th and 111th sessions (1993 and 2009), to center-right in the 105th–110th sessions (1997–2007).

### Coalition effectiveness

**Figure 4:**
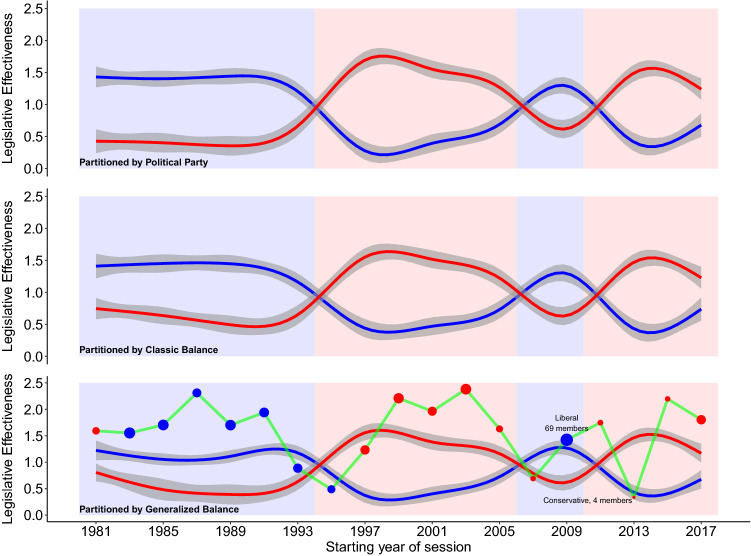
Mean of coalition members’ legislative effectiveness in the US House of Representatives. Blue (red) lines indicate the mean legislative effectiveness of Democrats (Republicans) in the top panel and that of the dominant liberal (conservative) coalitions in the center and bottom panels. In the bottom panel, the green line indicates the mean ideological effectiveness of members of the smallest coalition, while the size and color of the dot indicates the size and mean ideology of this coalition. Background shading indicates whether Democrats (blue) or Republicans (red) held a majority in the chamber during the respective session.

The primary task of legislators is to pass laws, and their ability to do so is referred to as *legislative effectiveness*^42–44^. Therefore, we examine the legislative effectiveness of coalitions in the US House of Representatives, again considering coalitions defined from three perspectives: party, classic balance ($k=2$), and generalized balance ($k=3$). Figure 4 displays coalition members’ mean effectiveness, for each method of defining coalitions (see *SI* Table S2). The left-leaning liberal coalition shown as a blue line and the right-leaning conservative coalition shown as a red line. Gray bands illustrate the 95% confidence interval around each estimate, while the blue (Democrat) and red (Republican) backgrounds indicate the majority party in a given session.

Coalitions based on political parties (Fig. 4, top panel) illustrate an expected pattern^45^: The majority party is most effective. This occurs not only because the majority party has more votes, but because it controls key procedural details of the chamber including deciding which bills will come for a vote and when (i.e. agenda-setting power^44^). Coalitions based on classic balance (Fig. 4, center panel) display essentially the same pattern.

Coalitions based on generalized balance (Fig. 4, bottom panel) also display a similar pattern, but with important differences. The large liberal coalition is still more effective when Democrats hold the majority, while the large conservative coalition is still more effective when Republicans hold the majority. However, these two dominant coalitions are both less effective than their party- or classic balance-defined counterparts. These lower levels of effectiveness are explained by the inclusion of the third coalition, shown as a green line, which is the most effective coalition in most sessions. The size and color of the dots along this green line indicate the third coalition’s size and median ideology, and highlight that members of the third coalition usually are ideologically aligned with the majority party.

During transitional periods when the majority party changed, members of the third coalition are temporarily less effective. However, during periods of stable party control^46^, the highly effective third coalition has been anchored by a small number of consistent and ultra-effective members. For example, the liberal-leaning third coalition during the Democratic-controlled 99th–102nd sessions (1985–1990) was anchored by Rep. Pat Williams (D-MT1, mean effectiveness = 4.49), Rep. Barney Frank (D-MA4, 4.02), and Rep. Daniel Glickman (D-KS4, 3.68). Similarly, the conservative-leaning third coalition during the Republican-controlled 106th–108th sessions (1999–2004) was anchored by Rep. Christopher Smith (R-NJ4, 8.44), Rep. Bill Young (R-FL10, 4.41), and Rep. Nancy Johnson (R-CT6, 2.98). Most recently, the conservative-leaning third coalition during the Republican-controlled 114th–115th sessions (2015–2018) was anchored by Rep. Edward Royce (R-CA39, 5.46), Rep. John Katko (R-NY24, 5.36), and Rep. Dave Reichert (R-WA8, 2.30).

Not only are members of the third coalition more effective than their traditional liberal and conservative coalition counterparts, but they also maintain distinctive political relations. Members of the traditional coalitions have 2.68 negative edges for every positive edge, but members of the third coalition have 21.18 negative edges for every positive edge (see *SI* Figure S3). Moreover, although 8.44% of traditional coalition members’ negative edges are with co-partisans (members of their own party), over one-quarter (25.6%) of third coalition members’ negative edges are with co-partisans.

## Discussion

Optimally partitioning signed networks according to generalized balance theory is computationally challenging, but often essential to understanding their structure. In this paper, we have developed a solution to this challenge, both demonstrating its computational feasibility and highlighting the novel structural insights that the resulting optimal partitions can reveal. Specifically, we have developed a pair of optimization models that make it practical to partition a signed network into exactly *k* clusters that minimize the number of frustrated edges across all possible *k*-partitions (taking 3.3 h on average for our networks with up to $\sim$ 30,000 edges using Eq. (1)), and to identify the smallest number of clusters that minimizes the number of frustrated edges across all possible partitions (taking 14 h on average for our networks with up to $\sim$ 30,000 edges using Eq. (2)). Applying these models to signed networks of collaboration and opposition among legislators in the US House allowed us to determine that these relationships are not structured by legislators’ political party affiliations, but instead by a three coalition system composed of a dominant liberal coalition, a dominant conservative coalition, and a previously obscured ‘third coalition’. This hidden third coalition is noteworthy because its median ideology is unstable, however its members are consistently more effective at passing legislation than their colleagues in either of the dominant coalitions.

Just as community detection algorithms advanced the ability to uncover patterns in unsigned networks a decade ago^28^, these models can advance the ability to uncover patterns in signed networks. However, unlike most community detection algorithms for which global optimization is not possible^47^, our models guarantee an optimal signed network partition. These innovations are important because signed networks are already studied in a wide range of contexts including biology^1–3^, finance^2,4^, and politics^5,7,20^. Moreover, statistical models now exist that enable signed networks to be constructed from virtually any empirical bipartite network data^48^, making signed networks available for analysis in a still broader range of contexts. The models we propose are perfectly general, but we demonstrated their practicality for globally optimal partitioning of real-world signed networks with up to 30,000 edges. In practice, this is a minor limitation because many empirical signed networks contain fewer edges, and models for constructing signed networks include methods for sparsifying otherwise dense signed networks^48^.

In addition to the methodological advances that our optimization models offer in the study of signed networks, our illustrative application of these models has also revealed a new way of thinking about how the US House of Representatives is organized. We observe that partitioning legislators into three coalitions according to generalized balance offers a better fit to their observed pattern of collaborations and oppositions than simply clustering them by political party. This suggests that the forces guiding coalition formation in the US House are more subtle and go beyond partisanship alone, even during periods of extreme polarization.

The previously obscured ‘third coalition’ we identified is unique in two important respects. First, members of the third coalition are highly effective at passing legislation, which has implications for how a party’s majority status is interpreted. Although members of the majority political party always appear to be more effective than members of the minority party, a substantial portion of this apparent majority advantage is conferred by the highly effective members of the third coalition, who tend to be ideologically aligned with the majority. Second, members of the third coalition have a much higher ratio of oppositions (negative edges) to collaborations (positive edges), and maintain more oppositions with members of their own party, which has implications for how membership in the third coalition is interpreted. These patterns suggest that although members of the third coalition may be ideologically aligned with the dominant coalition and majority party, they nonetheless represent a breakaway faction that are highly effective despite their rejection of partisanship. Our ability to identify such a cluster is noteworthy because it provides empirical support for earlier simulation studies suggesting that the introduction of independent legislators to an existing two-party legislature can increase the body’s overall legislative effectiveness^49^. Although these simulation studies might have been viewed as hinting at a strategy for reinvigorating democratic systems plagued by partisanship, our findings suggest it may already be in place in the US House of Representatives.

## Methods

We infer the collaboration and opposition patterns of legislators from their bill co-sponsorships^5,50,51^. These data begin as a bipartite network **B** in which legislators are connected to the bills they sponsor in a given session. From this, we construct the bipartite projection **P**, which captures the number of bills each pair of legislators has co-sponsored together. Finally, we use the Stochastic Degree Sequence Model (SDSM)^51^, implemented in the backbone package (version 1.5.0) in R^48,52^, to statistically infer a signed network of political collaboration and opposition. The SDSM applies a statistical test to the bipartite projection to yield a signed backbone **P**$'$ in which there exists a positive (negative) edge between each pair of legislators who have co-sponsored statistically significantly more (fewer) bills than expected by chance. The random expectation is obtained from a canonical null model in which bill sponsorship is random, but expected values of both degree sequences of **B** are preserved. Because the SDSM involves performing a statistical test for each pair of legislators, we ensure a family-wise error rate of $\alpha =0.01$ by applying a Holm-Bonferroni correction^53^.

We measure legislators’ ideology using 1^st^ dimension Nokken-Poole ideology scores obtained from the *Voteview database*^54^. These scores are similar to the widely used DW-Nominate ideological scores^55–57^, ranging from $-1$ (liberal) to 1 (conservative), except that they can vary across sessions. We measure legislators’ effectiveness using legislative effectiveness scores provided by the Center for Effective Lawmaking at https://thelawmakers.org/data-download. These scores were computed from fifteen indicators constructed from the intersection of three types of bills (commemorative, substantive, or substantive and significant) and five stages of a bill’s progression through the legislative life cycle (sponsored, committee action, post-committee action, chamber passage, and becoming law)^44^. These fifteen indicators capture the effectiveness of a legislator to advance their agenda items, and are normalized so that the mean effectiveness in each session is 1.

## Supplementary Information.

Supplementary Information.

Supplementary Dataset.

Supplementary Movie.

## Data Availability

All the data and codes used in this study are publicly available with links and descriptions provided in the Supplementary Information.
