# Supplementary material for: Identifying hidden coalitions in the US House of Representatives by optimally partitioning signed networks based on generalized balance
Source: Sci Rep. 2021 Oct 7;11:19939. doi: 10.1038/s41598-021-98139-w (PMC8497621; doi:10.1038/s41598-021-98139-w)
Supplement: Supplementary file 1 — Supplementary Information. [file 41598_2021_98139_MOESM1_ESM.pdf]

Supplementary information for identifying hidden coalitions in the US House of Representatives by optimally partitioning signed networks based on generalized balance

Samin Aref[1,2*] and Zachary P. Neal[3]

[1]Max Planck Institute for Demographic Research, 18057 Rostock, Germany
[2]Department of Mechanical and Industrial Engineering, University of Toronto, Toronto, ON M5S3G8, Canada
[3]Department of Psychology, Michigan State University, East Lansing, MI 48824, USA

*To whom correspondence should be addressed; E-mail: aref@mie.utoronto.ca

This PDF file includes:
Supplementary Text
Figs. S1 to S3
Tables S1 to S2
Caption for Movie S1
Captions for Databases S1 to S2
Supplementary References

Other Supplementary Materials for this manuscript include the following:
Movie S1
Databases S1 to S2

Supplementary Text

Data and code availability

All network data, numerical results, and replication code related to this study are publicly available with links provided in this document. The R code and the processed data for analyzing and visualizing the results are publicly available on an OSF repository at `https://doi.org/10.17605/OSF.IO/3QTFB`.

Solving the graph optimization models

The proposed optimization models can be solved by mathematical programming solvers which supports 0/1 linear programming (binary linear) models. The code for both optimization models will be made available on a GitHub repository at `https://github.com/saref/clusterability-index` once this paper is published. In the GitHub repository, we provide Python code for using Gurobi solver (version 9.1) to solve the proposed binary linear models and obtain optimal partitions of signed networks into internally cohesive and mutually divisive clusters based on generalized balance theory.

An illustrative numerical example for the k-partitioning model

We provide a numerical example to illustrate how the mathematical programming model in Eq. 1 (in the paper) works (and how it is solved by a branch and bound algorithm). Consider that the model in Eq. 1 is given the example signed graph of Fig. 1 (in the paper) and a the pre-defined value of $k = 3$ for the number of clusters.

The main role of the solver that solves this model is to explore the space of feasible solutions (feasible ways of clustering the input signed graph into k clusters) and finding a feasible solution which is associated with the minimum number of frustrated edges. Without loss of generality, we can consider one step of this optimization process is evaluating the objective function value (the frustration count) for a given feasible solution. The following numerical example explains how the solver handles the model to complete this step and move forward if needed.

Consider that the optimization solver is to evaluate the frustration count of the partition illustrated in Fig. 1 (B). The non-zero x_{ic} binary decision variables for this partition are as follows: $x_{1,1} = 1$, $x_{2,1} = 1$, $x_{3,1} = 1$, $x_{4,2} = 1$, and $x_{5,2} = 1$. Every other x_{ic} variable has to be 0 for these variables to constitute a feasible solution (due to the first set of constraints of the model $\sum_{c \in C} x_{ic} = 1 \forall i \in V$).

The second and third sets of constraints allow the model to determine the frustration status of each edge by quantifying all f_{ij} variables based on the values of the x_{ic} variables for the feasible solution under evaluation.

For the positive edges $(1, 3)$ and $(2, 3)$, the second set of constraints ($f_{ij} \geq x_{ic} - x_{jc} \, \forall (i, j) \in E^+$, $\forall c \in C$) is in place. These constraints for the feasible solution in Fig. 1 (B) lead to $f_{i,j} \geq 0$. Given the flexibility for taking either binary value, the minimization pressure from the objective function sets the values for $f_{1,3}$ and $f_{2,3}$ to 0. This means that the edges $(1, 3)$ and $(2, 3)$ are not frustrated because they are positive and have the same cluster membership on their endpoints.

For the negative edges $(1, 4)$, $(1, 5)$, $(2, 5)$, and $(3, 4)$, the third set of constraints ($f_{ij} \geq x_{ic} + x_{jc} - 1 \, \forall (i, j) \in E^-$, $\forall c \in C$) is in place. These constraints for the feasible solution in Fig. 1 (B) lead to $f_{i,j} \geq 0$. Given the flexibility for taking either binary value, the minimization pressure from the objective function sets the values for $f_{1,4}$, $f_{1,5}$, $f_{2,5}$, and $f_{3,4}$ to 0. This means that the edges $(1, 4)$, $(1, 5)$, $(2, 5)$, and $(3, 4)$ are not frustrated because they are negative and have different cluster memberships on their endpoints.

For the edge $(4, 5)$, the third set of constraints is in place because it is a negative edge. The constraint associated with $c = 2$ leads to $f_{i,j} \geq 1$ for the feasible solution in Fig. 1 (B). Therefore, $f_{4,5}$ takes the value 1. This means that the edge $(4, 5)$ is frustrated because it is negative and has the same cluster membership on its endpoints.

Accordingly, the objective function $\sum_{(i,j) \in E} f_{ij}$ is evaluated by the model to 1 for the partition illustrated in Fig. 1 (B). As the linear programming relaxation of the model in Eq. 1 has a solution of 0 for the signed graph in Fig. 1, the solver does not stop at this feasible solution and continues exploring other feasible solutions.

At some point, it finds the feasible solution for the partition illustrated in Fig. 1 (C). The constraints of the model and the pressure from the minimization objective function lead to all $f_{i,j}$ variables taking the value 0. Therefore, the objective function evaluates to 0.

At this stage of the branch and bound process, the upper bound (objective function of the best feasible solution found so far) and the lower bound (LP relaxation solution) reach each other and the solver stops and reports the partition illustrated in Fig. 1 (C) as an optimal k-partition for the input signed graph and the pre-defined parameter $k = 3$.

An illustrative numerical example for the partitioning model

We provide a numerical example to illustrate how the mathematical programming model in Eq. 2 (in the paper) works (and how it is solved by a branch and bound algorithm). Consider that the model in Eq. 2 is given the example signed graph of Fig. 1 (in the paper).

The main role of the solver that solves this model is to explore the space of feasible solutions (feasible ways of clustering the input signed graph into any number of clusters) and finding a feasible solution which is associated with the minimum number of frustrated edges. Without loss of generality, we can consider one step of this optimization process is evaluating the objective function value (the frustration count) for a given feasible solution. The following numerical example explains how the solver handles the model to complete this step and move forward if needed.

Consider that the optimization solver is to evaluate the frustration count of the partition illustrated in Fig. 1 (B). The non-zero y_{ij} binary decision variables for this partition are as follows: $y_{1,2} = 1$, $y_{1,3} = 1$, $y_{2,3} = 1$, and $y_{4,5} = 1$. Every other y_{ij} is 0 because no other pairs of nodes are in the same cluster.

Note that the term in the objective function for a positive edge is $1 - y_{ij}$ because a positive edge is frustrated when its endpoints are in different clusters. The term in the objective function for a negative edge is y_{ij} because a negative is frustrated when its endpoints are in the same cluster.

Given the values of $y_{1,3} = 1$ and $y_{2,3} = 1$, the contribution of positive edges $(1, 3)$ and $(2, 3)$ to the objective function is 0. This means that the positive edges $(1, 3)$ and $(2, 3)$ are not frustrated because they have the same cluster membership on their endpoints in the partition illustrated in Fig. 1 (B).

Given the value of $y_{4,5} = 1$, the negative edge $(4, 5)$ contributes 1 to the objective function. This means that the negative edge $(4, 5)$ is frustrated because it has the same cluster membership on its endpoints. The contribution of all other negative edges is 0 because they all have different cluster memberships on their endpoints in the partition illustrated in Fig. 1 (B).

Accordingly, the objective function $\sum_{(i,j) \in E} a_{ij}((a_{ij} + 1)/2) - a_{ij} y_{ij}$ is evaluated by the model to 1 for the partition illustrated in Fig. 1 (B). As the linear programming relaxation of the model in Eq. 2 has a solution of 0 for the signed graph in Fig. 2, the solver does not stop at this feasible solution and continues exploring other feasible solutions.

At some point, it finds the feasible solution for the partition illustrated in Fig. 1 (C). The non-zero y_{ij} binary decision variables for this partition are as follows: $y_{1,2} = 1$, $y_{1,3} = 1$, and $y_{2,3} = 1$. Every other y_{ij}

is 0 because no other pairs of nodes are in the same cluster. The objective function evaluates to 0 because all positive edges have the same cluster membership on their endpoints and all negative edges have different cluster memberships on their endpoints.

At this stage of the branch and bound process, the upper bound (objective function of the best feasible solution found so far) and the lower bound (LP relaxation solution) reach each other and the solver stops and reports the partition illustrated in Fig. 1 (C) as an optimal partition for the input signed graph.

Using Gurobi for solving the proposed optimization models

Our proposed algorithms are developed in Python 3.8 based on the mathematical programming models discussed in the paper which partition signed networks based on generalized balance into an optimal k-partition or an optimal partition without specifying k.

These optimization algorithms are distributed under an Attribution-NonCommercial-ShareAlike 4.0 International (CC BY-NC-SA 4.0) license. This means that one can use these algorithms for non-commercial purposes provided that they provide proper attribution for them by citing the current article. Copies or adaptations of the algorithms should be released under the similar license.

The following steps outline the process for academics to install the required software (*Gurobi* solver [1]) on their computer to be able to solve the optimization models:

1. Download and install Anaconda (Python 3.8 version) which allows you to run a Jupyter code. It can be downloaded from `https://anaconda.com/products/individual`. Note that you must select your operating system first and download the corresponding installer.

2. Register for an account on `https://pages.gurobi.com/registration` to get a free academic license for using Gurobi. Note that Gurobi is a commercial software, but it can be registered with a free academic license if the user is affiliated with a recognized degree-granting academic institution. This involves creating an account on Gurobi website to be able to request a free academic license in step 5.

3. Download and install Gurobi Optimizer (versions 9.1 and above are recommended) which can be downloaded from `https://www.gurobi.com/downloads/gurobi-optimizer-eula/` after reading and agreeing to Gurobi's End User License Agreement.

4. Install Gurobi into Anaconda. You do this by first adding the Gurobi channel to your Anaconda channels and then installing the Gurobi package from this channel.

   From a terminal window issue the following command to add the Gurobi channel to your default search list

   ```
   conda config --add channels http://conda.anaconda.org/gurobi
   ```

   Now issue the following command to install the Gurobi package

   ```
   conda install gurobi
   ```

5. Request an academic license from `http://gurobi.com/downloads/end-user-license-agreement-academic/` and install the license on your computer following the instructions given on Gurobi license page.

Completing these steps is explained in the following links (for version 9.1).

For Windows:

`gurobi.com/documentation/9.1/quickstart_windows/index.html`

For Linux:

`gurobi.com/documentation/9.1/quickstart_linux/index.html`

For Mac OS:

`gurobi.com/documentation/9.1/quickstart_mac/index.html`.

After following the instructions above, open Jupyter Notebook which takes you to an environment (a new tab on your browser pops up on your screen) where you can open the main code in a Jupyter notebook (which is a file with .ipynb extension).

Visualization of 3-partition coalitions in selected House networks (Figures S1 and S2)

Fig. S1 shows the 3-partition coalitions in the 101st, when the third coalition was dominated by highly effective ideologically liberal legislators. Fig. S2 shows the 3-partition coalitions in the 108th, when the third coalition was dominated by highly effective ideologically conservative legislators. In both cases, brown (positive) and turquoise (negative) edges represent significantly many and significantly few co-sponsorships respectively. Node color indicates the legislator's ideology on a blue (liberal, Nokken-Poole = -1), purple (moderate, 0), red (conservative, +1) spectrum. Node size indicates the legislator's effectiveness. All nodes are labeled with legislators' names, which are visible when the figure is viewed at $400^{+}\%$ magnification.

Session 101

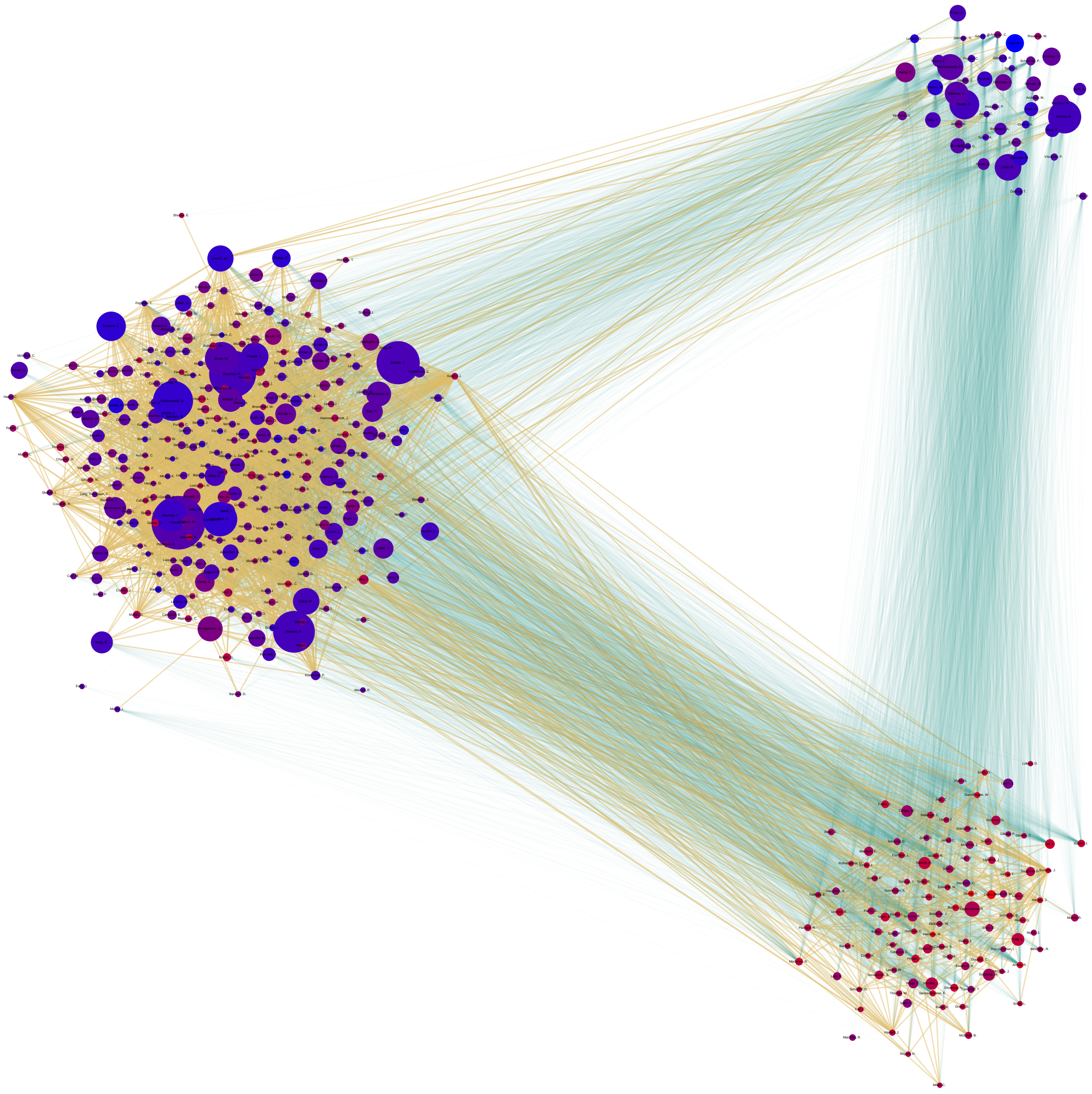

Figure S1: The 3-partition coalitions in the 101st session of the House of Representatives

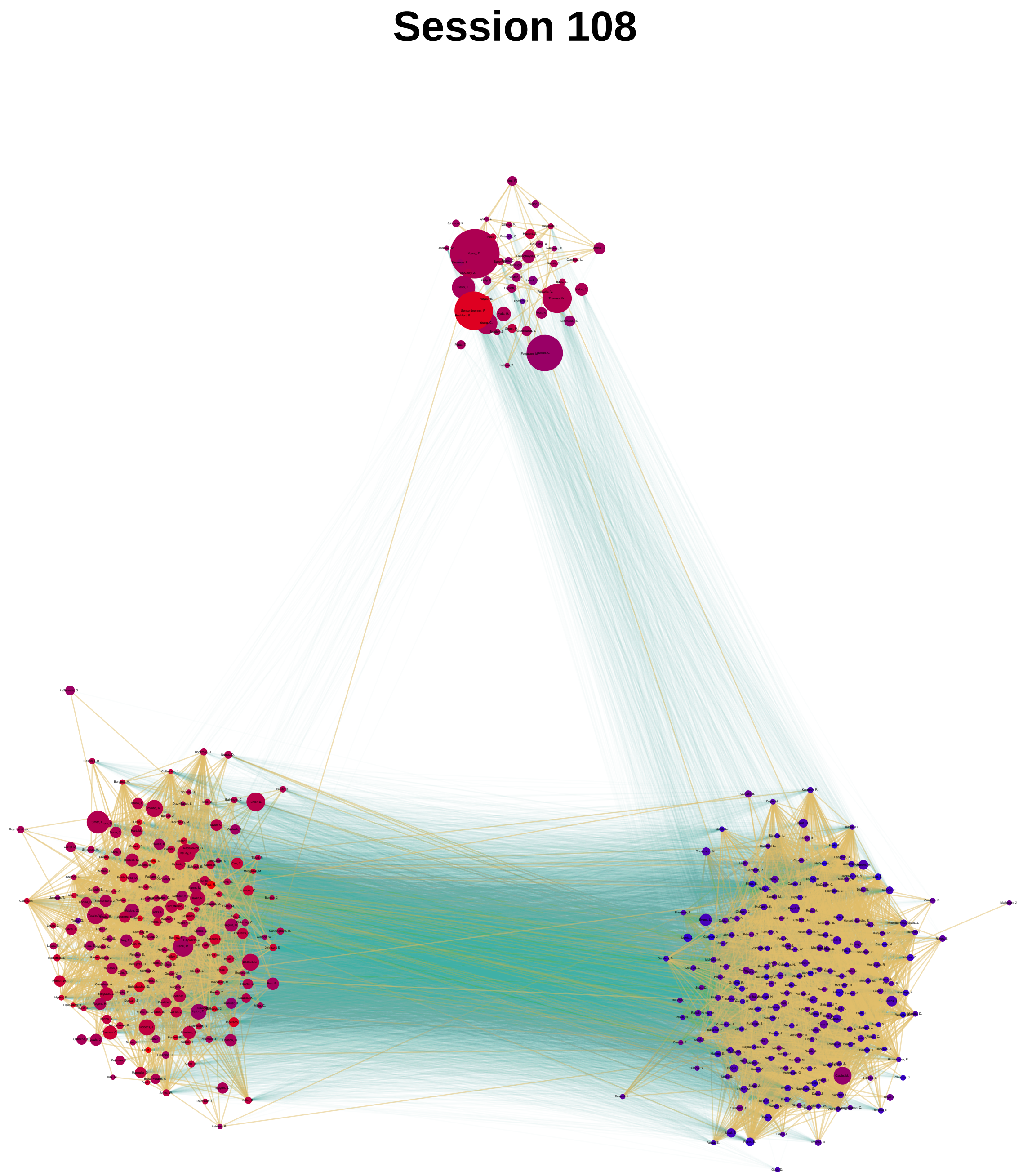

Figure S2: The 3-partition coalitions in the 108th session of the House of Representatives

Significance, generalizability, and limitations of our computational methods

The computational results we provided have broad relevance because they demonstrate the practical feasibility of solving fundamental NP-hard signed graph partitioning problems. Solving these partitioning problems are essential for exact evaluations of the structure of signed networks which go beyond the political science application we have demonstrated and have use cases in other fields from biology and physics [2, 3, 4, 5, 6, 7] to social sciences [8, 9, 10, 11, 12, 13, 14, 15, 16]. Specifically, our methods for partitioning a signed graph according to generalized balance improve upon heuristic methods that are fast but do not generally yield optimal partitions [17, 18, 19]. Additionally, our methods also improve upon existing methods for obtaining optimal partitions that are only capable of handling small graphs with $n \leq 40$ [20, 21]. The correctness of our methods for partitioning signed networks is guaranteed by the branch and bound algorithm of Gurobi [1] which is an exact method for solving binary linear programming models to global optimality.

The sizes of real-world instances we have solved to global optimality are considerable and therefore suggest that our proposed models can be used for a wide variety of other applications with networks of similar and smaller sizes. For example, the network of the 115th session has $n = 448$ nodes, $m = 31,936$ edges, and $|T| = 14,885,696$ connected triads. Obtaining an optimal 7-partition using Eq. 1 leads to an optimization model with $nk + m = 35,072$ binary variables and $mk + n = 224,000$ constraints, which takes Gurobi, 1.66 hours to solve. Moreover, obtaining an optimal partition (without specifying k) using Eq. 2 leads to an optimization model with $n(n-1)/2 = 100,128$ binary variables and $3|T| = 44,657,088$ constraints, which takes Gurobi only 5.28 hours to solve. While obtaining these partitions requires a few hours, the resulting partition is guaranteed to be globally optimal, which is essential for an exact evaluation of the structure of the signed networks under analysis.

As expected from the NP-hardness of the problems, the main limitation of the models in Eqs. 1–2 (in the manuscript) is the size of the network they can handle in a reasonable time. We have demonstrated the practicability of these models for real-world political networks with up to $\sim 30,000$ edges considering that a few hours is worth finding a globally optimal solution for the exact evaluation of the structure of these network. From a practical standpoint, two factors are relevant for determining whether these computationally intensive models are suitable for a different use case: network properties and processing capabilities. Previous studies suggest that some properties of the input graph like degree heterogeneity could be determinant factors of solve time in similar problems [22]. Also, structural regularities in networks constructed from empirical data often make them easier to solve compared to synthetic networks (like random graphs) [22]. As Gurobi solver makes use of multiple processing threads to explore the feasible space in parallel, the processing capabilities of the computer that runs the optimization solver could also make an impact. Therefore, our experiments do not guarantee that every network with up to $30,000$ edges can be optimally partitioned based on generalized balance within the solve times that we have observed for our real-world instances of US House signed networks.

The computing processor configuration we have used (32 Intel Xeon CPU E7-8890 v3 @ 2.50 GHz processors) and the size of the real networks we have analyzed ($m \sim 30,000$) have led to solve times of roughly a few hours per instance. One could speculate that larger networks on the same hardware or the same networks on less powerful hardware is expected to take longer. In such cases, one may consider using a non-zero optimality gap tolerance (*MIPGap* as a Gurobi parameter [1]) to find solutions within a guaranteed proximity of optimality to reduce the solve time.

Multiplicity of optimal solutions

There are symmetries in the mathematical formulations for the two models in Eqs. 1–2 (in the manuscript). For example, in Eq. 1, a given 2-partition can be expressed by different feasible solutions (sets of values for

decision variables). This is because the clusters are treated indifferently and could be swapped while the partition remains virtually unchanged. As another example, in Eq. 2, a feasible solution does not necessarily represent a unique partition. This is due to the original formulation [23] in which a pair of non-adjacent nodes a and i may have no decision variables indicating they belong to the same cluster with any of their neighbours (denoted by b and j respectively $\forall b, j : a_{ab} \neq 0, a_{ij} \neq 0$), i.e., all the decision variables associated with a and i take the value zero. In that case, the same feasible solution could lead to two partitions (with identical fitness) depending on whether nodes a and i are placed in the same or different clusters. Another source of symmetry is the existence of isolate nodes whose optimal cluster membership is random and therefore not meaningful. When characterizing the composition of clusters in our analyses, we have ignored isolates.

Due to the symmetries outlined above, both optimization models in Eqs. 1–2 generally have multiplicity in their optimal solutions. Finding all optimal solutions to such computationally intensive problems are not practically feasible for large instances. For small instances, however, previous studies have looked at multiplicity of optimal solutions in similar partitioning problems [15, 24]. Although optimal 2-partitions can be unique in some small real-world signed networks [15], more often multiple optimal solutions exist [15, their Fig. S1]. Also, in the case of small complete random signed graphs, multiple optimal solutions may exist [24]. Due to the practical complexity of these problems and the size of empirical networks we consider, although we cannot find and analyze all optimal partitions, it is certain that the optimal partitions are not unique. Future work is needed to find practical methods for finding and analyzing all optimal partitions of such large networks.

Oppositional ties of the splinter coalition

Members of the third coalition have 21.18 negative ties for every positive tie which is substantially different from the members of traditional coalitions who have on average 2.68 negative ties for every positive tie. This distinction in oppositional ties deserves more attention and we look at the fraction of each type of edge by coalition, taking into account the party of legislator at the other endpoint of the edge.

Figure S3 illustrates the fractions of positive and negative edges with co-partisans (members of the same party) for each of the coalitions based on the optimal 3-partitions. Fractions of positive (negative) edges are shown by solid (dashed) lines. The red, blue, and green lines represent the conservative coalition, the liberal coalition, and the splinter coalition respectively. It can be seen in Figure S3 that the three coalitions are similar based on the fraction of positive edges with co-partisans: members of all coalitions mainly collaborate (i.e. have a positive edge with) members of their own party. For the fraction of negative edges with co-partisans, however, the splinter coalition shifts away from the main liberal and conservative coalition. From the 104th session, this quantity has generally increased for the splinter coalition reaching values close to 0.4. This means that legislators in the splinter coalitions have a considerable proportion (nearly 40%) of their negative edges with members of the own party. Given this distinctive feature in oppositional ties, one may conclude that the members of the third coalition are distinctively more willing to push back against their own party.

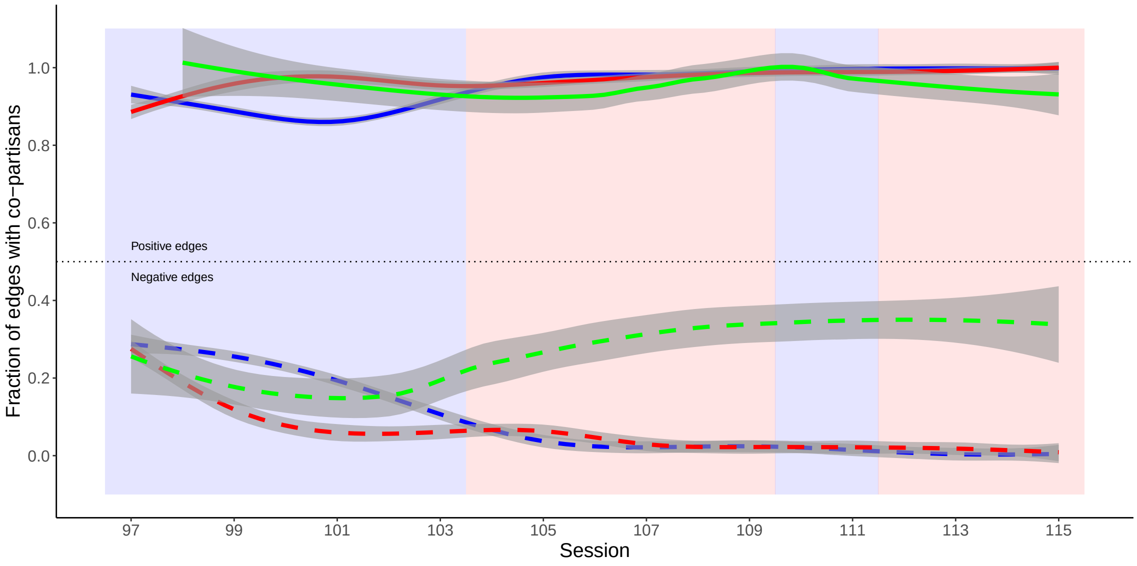

Figure S3: Fractions of positive and negative edges to members of the same party aggregated for each of the three coalitions

Additional numerical results (Tables S1 to S2)

Table S1: Detailed properties and clusterability indices for networks

Session	Start year	n	m	density	m^-	m^+	m^-/m	$C_2(G)$	$C_3(G)$	$C_4(G)$	$C_5(G)$	$C_6(G)$	$C_7(G)$	$C(G)$
97	1981	447	4954	0.05	4494	460	0.91	280	36	14	6	**4**	**4**	**4**
98	1983	444	6744	0.07	5460	1284	0.81	435	102	60	49	45	**44**	**44**
99	1985	443	8714	0.07	6759	1955	0.78	962	79	48	40	**38**	**38**	**38**
100	1987	446	8520	0.08	6888	1632	0.81	560	118	89	**84**	**84**	**84**	**84**
101	1989	449	10180	0.10	7970	2210	0.78	896	263	207	188	**183**	**183**	**183**
102	1991	447	13692	0.11	10152	3540	0.74	748	169	119	112	**111**	**111**	**111**
103	1993	446	13281	0.10	10530	2751	0.79	301	245	**241**	**241**	**241**	**241**	**241**
104	1995	445	13019	0.13	10301	2718	0.79	17	**7**	**7**	**7**	**7**	**7**	**7**
105	1997	449	16709	0.13	13125	3584	0.79	50	22	**16**	**16**	**16**	**16**	**16**
106	1999	442	17746	0.14	13390	4356	0.75	91	48	40	**39**	**39**	**39**	**39**
107	2001	447	17908	0.14	14062	3846	0.79	59	34	**31**	**31**	**31**	**31**	**31**
108	2003	444	19384	0.16	14211	5173	0.73	63	33	**29**	**29**	**29**	**29**	**29**
109	2005	445	21601	0.17	15684	5917	0.73	69	44	35	**34**	**34**	**34**	**34**
110	2007	452	23719	0.15	17094	6625	0.72	44	31	**30**	**30**	**30**	**30**	**30**
111	2009	451	21564	0.21	15342	6222	0.71	153	87	82	**78**	**78**	**78**	**78**
112	2011	450	29904	0.19	21118	8786	0.71	39	30	**28**	**28**	**28**	**28**	**28**
113	2013	447	25992	0.21	19026	6966	0.73	24	20	**18**	**18**	**18**	**18**	**18**
114	2015	446	30149	0.22	21299	8850	0.71	57	38	**37**	**37**	**37**	**37**	**37**
115	2017	448	31936	0.32	21883	10053	0.69	221	38	**35**	**35**	**35**	**35**	**35**

Cases indicating the stagnation of frustrated edges are shown in bold-face font.

Table S2: The size and effectiveness of the two parties and optimal 3-partition coalitions

	Partition based on political party						Generalized Balance Partition ($k = 3$)								
	Size		Median Ideology		Mean Effectiveness		Size			Median Ideology			Mean Effectiveness		
session	D	R	D	R	D	R	L	C	S	L	C	S	L	C	S
97	245	194	-0.33	0.28	1.45	0.44	153	255	18	-0.40	0.20	0.29	1.29	0.83	1.59
98	272	167	-0.32	0.32	1.37	0.39	288	99	51	-0.22	0.32	-0.37	1.05	0.55	1.55
99	256	182	-0.32	0.34	1.45	0.37	304	85	47	-0.19	0.41	-0.41	1.07	0.40	1.70
100	261	179	-0.33	0.35	1.40	0.43	282	125	29	-0.28	0.39	-0.34	1.09	0.54	2.31
101	264	179	-0.33	0.35	1.41	0.40	290	105	45	-0.22	0.42	-0.37	1.10	0.40	1.70
102	270	170	-0.33	0.34	1.40	0.39	299	100	37	-0.24	0.41	-0.36	1.11	0.42	1.94
103	259	180	-0.34	0.38	1.45	0.32	224	176	32	-0.36	0.38	-0.21	1.53	0.35	0.89
104	207	231	-0.38	0.38	0.33	1.60	140	258	22	-0.44	0.36	-0.31	0.31	1.39	0.49
105	211	231	-0.39	0.39	0.33	1.62	194	208	32	-0.40	0.41	0.15	0.40	1.50	1.23
106	212	224	-0.39	0.39	0.39	1.58	210	182	42	-0.39	0.43	0.23	0.41	1.41	2.21
107	213	228	-0.39	0.38	0.41	1.54	204	202	30	-0.39	0.40	0.24	0.44	1.42	1.96
108	208	230	-0.39	0.41	0.40	1.53	205	186	46	-0.39	0.43	0.29	0.43	1.27	2.38
109	203	236	-0.38	0.41	0.48	1.51	210	210	15	-0.38	0.42	0.25	0.48	1.46	1.63
110	241	206	-0.38	0.43	1.45	0.45	242	195	8	-0.38	0.44	0.12	1.43	0.44	0.69
111	263	182	-0.37	0.47	1.36	0.44	195	176	69	-0.42	0.47	-0.18	1.34	0.44	1.42
112	198	245	-0.40	0.47	0.47	1.42	178	250	9	-0.41	0.46	0.29	0.47	1.33	1.75
113	203	238	-0.40	0.48	0.51	1.42	197	236	4	-0.41	0.48	0.06	0.52	1.43	0.34
114	188	249	-0.40	0.49	0.58	1.32	186	242	8	-0.40	0.50	0.21	0.58	1.28	2.20
115	195	243	-0.39	0.49	0.59	1.35	192	211	33	-0.39	0.52	0.24	0.59	1.27	1.80

In party-partition: D = Democratic coalition, R = Republican coalition

In 3-partition: L = Liberal coalition, C = Conservative coalition, S = Splinter coalition

Movie: Slideshow of the 3-partition coalitions of the signed US House networks

A slideshow of optimal 3-partition coalitions is available online at `https://saref.github.io/SI/AN2021/House_coalitions.mp4` which includes all 19 House networks. Green and red edges represent significantly many and significantly few co-sponsorships respectively. Node color indicates the legislator's ideology on a blue (liberal, Nokken-Poole = -1), purple (moderate, 0), red (conservative, +1) spectrum. Node size indicates the legislator's effectiveness. Looking at the colors and positions of edges we can see that the large majority of edges are intra-cluster positive or inter-cluster negative. In these networks, only 0.05%–2.5% of the edges are frustrated under the optimal 3-partitions which indicate the closeness of the networks to the assertions of the generalized balance theory [25]. If we look at the colors of the nodes, we see the ideological divide between the members of different coalitions. The splinter coalition is the smallest cluster of the nodes which usually has several large nodes (highly effective legislators).

Dataset: frustrated_legislators.RData and frustrated_legislators.R on OSF

The file 'frustrated_legislators.RData' is an R workspace which includes a dataframe object 'data' that contains details about each legislator in each session (e.g. ideology, effectiveness, cluster membership in optimal k-partitions), and 19 igraph objects 'H###' that contain signed networks for each session. The file 'frustrated_legislators.R' in the same repository contains the R code to replicate all substantive analyses reported in the manuscript using these data. Both files are publicly available at `https://doi.org/10.17605/OSF.IO/3QTFB`. The data are distributed under a CC-BY 4.0 license, which means that they can be used provided they are properly attributed by citing [12, 26] and the current article.

Dataset: clusters-house.csv

The results on globally optimal solutions to the optimization model for k-partitioning House networks are available in comma-separated values format at `saref.github.io/SI/AN2021/clusters-house.csv`. The first and second columns contain session numbers and legislator name as indicated by the headers. Each row is a legislator-session combination. The other columns are the cluster assignments based on optimal k-partitions for $k \in \{2, 3, \dots, 7\}$ as indicated by the column header. The entries represent the cluster assignment of the node associated to the row (the legislator-session combination) based on an optimal solution of the k-partition associated to the column.

References

[1] Gurobi Optimization Inc. Gurobi optimizer reference manual, 2020. url: `http://gurobi.com/documentation/9.1/refman/index.html` date accessed 1 December 2020.

[2] Giovanni Iacono, Fahimeh Ramezani, Nicola Soranzo, and Claudio Altafini. Determining the distance to monotonicity of a biological network: a graph-theoretical approach. *IET Systems Biology*, 4(3):223–235, 2010.

[3] Falk Hüffner, Nadja Betzler, and Rolf Niedermeier. Separator-based data reduction for signed graph balancing. *Journal of Combinatorial Optimization*, 20(4):335–360, 2010.

[4] Giuseppe Facchetti, Giovanni Iacono, and Claudio Altafini. Computing global structural balance in large-scale signed social networks. *Proceedings of the National Academy of Sciences*, 108(52):20953–20958, 2011.

[5] Giuseppe Facchetti, Giovanni Iacono, and Claudio Altafini. Exploring the low-energy landscape of large-scale signed social networks. *Physical Review E*, 86(3):036116, 2012.

[6] Amirhessam Tahmassebi, Behshad Mohebali, Lisa Meyer-Baese, Philip Solimine, Katja Pinker, and Anke Meyer-Baese. Determining driver nodes in dynamic signed biological networks. In Brian M. Cullum, Douglas Kiehl, and Eric S. McLamore, editors, *Smart Biomedical and Physiological Sensor Technology XVI*, volume 11020, pages 53 – 60. International Society for Optics and Photonics, SPIE, 2019.

[7] Lingbo Li, Ying Fan, An Zeng, and Zengru Di. Binary opinion dynamics on signed networks based on Ising model. *Physica A: Statistical Mechanics and its Applications*, 525:433–442, 2019.

[8] Frank Harary, Meng-Hiot Lim, and Donald C. Wunsch. Signed graphs for portfolio analysis in risk management. *IMA Journal of Management Mathematics*, 13(3):201–210, 2002.

[9] Patrick Doreian and Andrej Mrvar. Structural Balance and Signed International Relations. *Journal of Social Structure*, 16:1–49, 2015.

[10] Pedro C Souto, Andreia Sofia Teixeira, Alexandre P Francisco, and Francisco C Santos. Capturing financial volatility through simple network measures. In *International Conference on Complex Networks and their Applications*, pages 534–546, Cham, Switzerland, 2018. Springer.

[11] Tyler Derr and Jiliang Tang. Congressional vote analysis using signed networks. In *2018 IEEE International Conference on Data Mining Workshops (ICDMW)*, pages 1501–1502. IEEE, 2018.

[12] Zachary P. Neal. A sign of the times? Weak and strong polarization in the U.S. Congress, 19732016. *Social Networks*, 60:103 – 112, 2020.

[13] Samin Aref and Zachary P. Neal. Detecting coalitions by optimally partitioning signed networks of political collaboration. *Scientific Reports*, 10(1):1–10, 2020.

[14] Nejat Arınık, Rosa Figueiredo, and Vincent Labatut. Multiple partitioning of multiplex signed networks: Application to European parliament votes. *Social Networks*, 60:83–102, 2020.

[15] Samin Aref, Ly Dinh, Rezvaneh Rezapour, and Jana Diesner. Multilevel structural evaluation of signed directed social networks based on balance theory. *Scientific Reports*, 10(1):1–12, 2020.

[16] David Schoch and Ulrik Brandes. Legislators roll-call voting behavior increasingly corresponds to intervals in the political spectrum. *Scientific Reports*, 10(1):1–9, 2020.

[17] Lúcia Drummond, Rosa Figueiredo, Yuri Frota, and Mário Levorato. Efficient solution of the correlation clustering problem: An application to structural balance. In Yan Tang Demey and Hervé Panetto, editors, *On the Move to Meaningful Internet Systems: OTM 2013 Workshops*, pages 674–683, Berlin, Heidelberg, 2013. Springer.

[18] Mario Levorato, Lucia Drummond, Yuri Frota, and Rosa Figueiredo. An ILS algorithm to evaluate structural balance in signed social networks. In Alessio Bechini and Jiman Hing, editors, *Proceedings of the 30th Annual ACM Symposium on Applied Computing*, SAC '15, pages 1117–1122, NY, USA, 2015. ACM.

[19] Mario Levorato, Rosa Figueiredo, Yuri Frota, and Lúcia Drummond. Evaluating balancing on social networks through the efficient solution of correlation clustering problems. *EURO Journal on Computational Optimization*, 5(4):467–498, 2017.

[20] Michael Brusco and Douglas Steinley. K-balance partitioning: An exact method with applications to generalized structural balance and other psychological contexts. *Psychological Methods*, 15(2):145–157, 2010.

[21] Rosa Figueiredo and Gisele Moura. Mixed integer programming formulations for clustering problems related to structural balance. *Social Networks*, 35(4):639–651, 2013.

[22] Samin Aref, Andrew J. Mason, and Mark C. Wilson. A modeling and computational study of the frustration index in signed networks. *Networks*, 75(1):95–110, 2020.

[23] Erik D Demaine, Dotan Emanuel, Amos Fiat, and Nicole Immorlica. Correlation clustering in general weighted graphs. *Theoretical Computer Science*, 361(2):172–187, 2006.

[24] Nejat Arınık, Rosa Figueiredo, and Vincent Labatut. Multiplicity and diversity: analysing the optimal solution space of the correlation clustering problem on complete signed graphs. *Journal of Complex Networks*, 8(6), 03 2021.

[25] James A Davis. Clustering and structural balance in graphs. *Human Relations*, 20(2):181–187, 1967.

[26] Rachel Domagalski, Zachary P. Neal, and Bruce Sagan. Backbone: An R package for extracting the backbone of bipartite projections. *PLOS One*, 16(1):1–20, 2021.
